# Welsh Hospital Managers

**Published:** 1911-12-23

**Authors:** 


### Welsh Hospital Managers.

### IMPORTANT ACTION BY COLONEL BRUCE VAUGHAN, CHAIRMAN OF KING EDWARD VII.'s HOSPITAL, CARDIFF.

At the monthly meeting of the Board of Management of King Edward VII.'s Hospital, Cardiff, Colonel Bruce Vaughan expressed misgivings as to the effects of the Insurance Bill upon hospital resources, and on the following evening, when addressing a meeting at Dowlais, Mr. Edgar Jones, M.P., took exception to Colonel Vaughan's remarks. Mr. Jones is reported as having said that the Bill provided, not in one, but in several clauses, for direct contributions on behalf of inmates of hospitals through the approved societies and the local health committees. The Bill must necessarily secure to hospitals a fixed, reliable, constant source of income which they could not draw upon at the present time. Mr. Jones's comments have drawn a reply from Colonel Bruce Vaughan, from which we take the following:—

"Mr. Edgar Jones says the Bill must necessarily secure to hospitals a fixed source of income that they could not draw at the present time."

Perhaps Mr. Edgar Jones will kindly name the clauses and explain the value of them to the hospitals and the insured patient, and where this fixed source of income is to come from which the hospitals do not enjoy at the present time.

If the Bill is to provide this fixed constant income it would be welcome news to those of us who have the responsibility of finding the income for the King Edward VII.'s Hospital.

But I am afraid the hon. member's words are misleading, for, in the opinion of the managers of 200 hospitals in the kingdom, it will do nothing of the kind.

Mr. Lloyd George's opinion seems to coincide with that of the managers, for he said to a deputation of them on July 29 last: "You should ask the patients, 'Are you insured?' If they were, then they had no right to come to hospitals."

Sir Henry Burdett says (and he has made a diligent search and study of the Bill) that there is no direct value in any one clause, but, on the contrary, the Insurance Bill does not provide the workmen of the United Kingdom with the hospital provision they at present enjoy, and gives them no substitute for it.

Mr. Edgar Jones's reply will be awaited with interest. He is a warm supporter of Mr. Lloyd George's policy, and between them they may be able to do what has proved impossible to all who have studied the Bill closely—i.e. to show how hospital managers can secure the rare and refreshing fruit which Mr. Edgar Jones believes the Act really offers to them for their enjoyment and upkeep. Failing this result, Mr. Edgar Jones will, we hope, join hands with us in working for an amending Act to protect the working classes throughout the country from the impending loss of hospital care when they most need it. The Bill as it stands creates the need for 30,000 hospital beds for the sole use of insured persons without providing one single hospital bed for their accommodation.

